# When More Is Better – Consumption Priming Decreases Responders’ Rejections in the Ultimatum Game

**DOI:** 10.3389/fpsyg.2017.02226

**Published:** 2017-12-20

**Authors:** Michael Zürn, Fritz Strack

**Affiliations:** ^1^Department of Psychology, Social and Economic Cognition II, University of Cologne, Cologne, Germany; ^2^Department of Psychology, University of Würzburg, Würzburg, Germany

**Keywords:** Ultimatum Game, comparison, consumption priming, evaluation, cognitive processes

## Abstract

During the past decades, economic theories of rational choice have been exposed to outcomes that were severe challenges to their claim of universal validity. For example, traditional theories cannot account for refusals to cooperate if cooperation would result in higher payoffs. A prominent illustration are responders’ rejections of positive but unequal payoffs in the Ultimatum Game. To accommodate this anomaly in a rational framework one needs to assume both a preference for higher payoffs and a preference for equal payoffs. The current set of studies shows that the relative weight of these preference components depends on external conditions and that consumption priming may decrease responders’ rejections of unequal payoffs. Specifically, we demonstrate that increasing the accessibility of consumption-related information accentuates the preference for higher payoffs. Furthermore, consumption priming increased responders’ reaction times for unequal payoffs which suggests an increased conflict between both preference components. While these results may also be integrated into existing social preference models, we try to identify some basic psychological processes underlying economic decision making. Going beyond the Ultimatum Game, we propose that a distinction between comparative and deductive evaluations may provide a more general framework to account for various anomalies in behavioral economics.

## Introduction

To understand human cooperation, it is not sufficient to rely on traditional economic theories of choice. While these theories (e.g., Friedman and Savage, 1948; von Neumann and Morgenstern, 1955) predict that people will cooperate as long as it maximizes their payoff, the Ultimatum Game (Güth et al., 1982), where two players only receive a payoff if they cooperate, illustrates that this is not always the case. In detail, this game is played between a proposer and a responder who negotiate how to split a given monetary “pie.” Proposers offer a certain payoff to responders who may either accept or reject it. If they accept, responders receive their agreed upon payoff and proposers receive the rest of the pie. Otherwise, no one receives anything. That is, only if the responder accepts the proposer’s offer, cooperation will be established and the pie can be eaten.

Traditional theories of rational choice assume that decision makers always prefer more money to less (e.g., Friedman and Savage, 1948). Therefore, they predict very high degrees of cooperation in the Ultimatum Game but also very unequal splits of the pie. Specifically, these theories assume that a rational responder will accept any non-zero offer because something small is still better than nothing at all (which would be the payoff if the offer would be rejected). Because a rational proposer will anticipate this behavior of the responder, the utility maximizing, i.e., rational choice is to offer the smallest non-zero payoff. In sum, Ultimatum Games between traditionally rational players will always end with the cooperative, mutually advantageous outcome where the proposer gets almost everything and the responder gets almost nothing. Nonetheless, numerous variations of the game have shown that human responders frequently reject non-zero offers if the split is unequal. Similarly, human proposers often do not dare to make the rational offer (Camerer, 2003; Güth and Kocher, 2014). Thus, the Ultimatum Game marks an anomaly (Thaler, 1988) because it challenges these traditional theories about human behavior.

Behavioral economists have accepted the challenge and have designed new theories that can manage cooperation in general and the Ultimatum Game in particular. Most prominently, the “social preference” approach has been used to “rationalize” the observable behavioral patterns in several economic games, including the Ultimatum Game (Bolton, 1991; Fehr and Schmidt, 1999; Bolton and Ockenfels, 2000; Charness and Rabin, 2002). Among each other, these models differ in important aspects but they all share the idea to extend the utility functions that underlie behavior. Specifically, “social preferences” include not only the traditional preference for higher payoffs but also a preference for equal payoffs. In the Ultimatum Game, responders have to trade these preference components off when they decide whether to accept or reject an unequal offer. That is, by rejecting such offers, responders can renounce a higher payoff if it opposes their preference for equality.

Throughout the history of economic thought, the preference for higher payoffs has been rooted in the consumption of goods purchased with the available income. For example, Friedman and Savage (1948, p. 288) state that “a higher income is desired because it enables a consumer unit to purchase a wider variety of commodities.” However, these authors further concluded that “[i]t simplifies matters, and involves no loss in generality, to regard the alternatives open to the consumer unit as capable of being expressed entirely in terms of money” (ibid). Correspondingly, economic games are traditionally played with monetary incentives. However, this simplification might unintentionally have affected preference structures in the Ultimatum Game. Specifically, the preference for higher payoffs may be attenuated relative to the preference for equal payoffs, if these payoffs are presented in monetary terms instead of consumption opportunities. That is, if responders recognize the consumptive potential inherent in the payoffs, the preference for higher payoffs might become stronger. Therefore, we hypothesize that responders reject fewer unequal payoffs if consumptive opportunities are salient. Put differently, responders then should be more likely to resolve the conflict (or trade-off) between the preference components in favor of their preference for higher payoffs. In contrast, proposer decisions are mainly determined by altruistic concerns and strategic considerations (Forsythe et al., 1994; Weg and Zwick, 1994). Therefore, the salience of consumptive opportunities should not affect proposer decisions. Importantly, however, our main hypothesis motivating this research concerns the effects of consumption priming on responder decisions.

In the current set of experiments, we use consumption priming to make consumptive opportunities more salient. In general, priming is a reliable way to increase the cognitive accessibility of specific concepts (see Strack and Schwarz, 2016). This psychological procedure has also been used in the domain of behavioral economics (see Cohn and Maréchal, 2016). For example, Posten et al. (2014) used priming in a Trust Game (Berg et al., 1995) and found that investors transferred more resources to a trustee if they were primed with trust than if they were primed with distrust. Moreover, Kay et al. (2004) primed Ultimatum Game proposers with the concept of competition to activate the related behavioral norms. As a consequence, proposers whose attention was directed toward competition made lower offers compared to participants who received no specific priming. Therefore, if responders are primed with consumption, the preference for higher payoffs should receive more weight in the decision. As a consequence, fewer unequal payoff distributions should be rejected.

## Experiment 1: Consumption Priming in the Ultimatum Game

In our first experiment, we directed participants’ attention toward the consumption opportunities their payoffs entail. As a consequence, the use value of the payoffs should become more salient which in turn should attenuate the characteristic anomaly of the Ultimatum Game. To some degree, we expected considerably stronger effects if the offers would create payoffs where the inequality was disadvantageous for the responders. After all, the aversion to advantageous inequality is considered significantly weaker than to disadvantageous inequality (see Fehr and Schmidt, 1999) which reduces the possibility that our manipulation can have an effect. That is, responders should accept more disadvantageously unequal offers if the offers’ consumption opportunities are primed. As outlined above, the intervention should not affect proposer decisions.

### Sample and Design

One hundred and fifteen participants (*age: M* = 25, *SD* = 7; 65% female) were recruited online for an experiment administered on their own devices. For this purpose, we used the SONA Systems mailing function to invite participants to an economic experiment. At the time of the data collection, the subject pool consisted of about 1500 people with a diverse demographic background. Because we did not know the size of the hypothesized effect in advance, we analyzed data from everyone who responded to our invitation. As a compensation for their efforts, participants took part in a lottery in which they could win a 50€ AMAZON gift voucher. The experiment took about 5 min.

Participants were tested in a 2 (role: proposer vs. responder, w/i) × 2 (prime: none vs. consumption, b/w) × 2 role order (proposer first vs. responder fist, b/w) mixed design. Each participant first played the Ultimatum Game in a randomly assigned role and then played a second, independent Ultimatum Game in the complementary role. Payoffs from both games were added up. The proposers’ offers and the responders’ decisions served as dependent variables.

### Materials and Procedure

First, participants were assured that they will not be deceived in this experiment and that all provided information (including the rules and consequences of the economic game) will be true. Next, we informed them that they would play a game with another participant in which they would negotiate about “tokens” that would later be exchanged for lottery tickets (exchange rate 1:1). We also explicitly pointed out that as a consequence, the more tokens they would earn in the game, the higher will be the probability for them to win the Amazon gift voucher. Moreover, we informed them that they would have to provide a valid email address to transfer this voucher in case they won. Naturally, we assured participants that the contact details will not be analyzed in any form and deleted afterward (see Zürn and Topolinski (2017) for a similar incentive structure).

The Ultimatum Game itself was largely similar in both experimental conditions. Before playing the first game, however, participants in the consumption-prime condition were reminded that they could win a gift voucher with the tokens earned in the game. To activate specific consumption opportunities, these participants had to contemplate for 1 min what they could buy with the gift voucher. Therefore, we presented them with the 10 main product categories offered by Amazon.de and asked them to select the category from which they would most likely buy something. Participants in the control condition directly proceeded to the following role assignment. All participants were randomly assigned either to the role of proposer or responder. Irrespective of their role, participants in the consumption-prime condition were then once more reminded of the Amazon voucher lottery and also of the Amazon product category they had chosen before. All proposers then had to indicate how many of the 10 available tokens they wanted to keep for themselves and how many they wanted to offer the responding participant. Responders’ choices were recorded using the strategy method (Selten, 1967; Fischbacher et al., 2001). That is, they had to indicate whether they would accept or reject each one of the 11 possible offers (0–10 tokens). The offers were presented in a list and had the format “X for you, Y for Player A.” After making their decision(s), participants in both roles proceeded to the second round where they played the opposite role.

At the end of both rounds, participants first indicated how often they had thought about purchasing something with the gift voucher during the game (1 = *not at all* – 6 = *all the time*) and answered demographical questions. After the data collection was complete, we randomly assigned each responder to a proposer and thereby determined the final responder decisions. The tokens were paid out according to the rules of the game and exchanged for lottery tickets. Afterwards, all lottery tickets were put in an urn and one ticket was randomly drawn. The email address on the ticket indicated the winner who was send the gift voucher by email. All steps, pairing of players, determining the payoffs and the lottery, were executed in an automatized process.

### Results

Our manipulation seems to have been successful because participants primed with consumption reported thinking significantly more about possible purchases (*M* = 2.07, *SE* = 0.13) than did participants in the control condition (*M* = 1.46, *SE* = 0.11), *t*(148) = 3.474, *p* < 0.001, *d* = 0.57.

#### Proposers^1^

The average offer made by the proposers was 4.52 tokens (*SE* = 0.09) which was significantly higher than one token, i.e., the smallest non-zero offer in our setting, *t*(149) = 39.285, *p* < 0.001. Moreover, a 2 prime (none vs. consumption) × 2 role order (proposer first vs. responder first) ANOVA yielded neither a significant effect of prime, *F*(1,146) = 0.171; *p* = 0.680; $\eta_{p}^{2}$ = 0.001, nor a significant effect of role order, *F*(1,146) = 1.236; *p* = 0.268; $\eta_{p}^{2}$ = 0.008. No significant interaction emerged, *F*(1,146) = 1.217; *p* = 0.272; $\eta_{p}^{2}$ = 0.008.

#### Responders

Based on our theorizing, we hypothesized that responders would accept more disadvantageously unequal offers if consumption is primed because the preference for higher payoffs is increased. Therefore, we first calculated the average acceptance rates of disadvantageously unequal offers (1 – 4 token offers) and then performed a one-tailed *t*-test tentatively suggesting that responders in the consumption prime condition (*M* = 0.54; *SE* = 0.04) accepted more disadvantageously unequal offers than responders in the control condition (*M* = 0.47; *SE* = 0.04), *t*(148) = 1.204, *p* = 0.12, *d* = 0.20. However, this difference was not statistically significant at conventional levels.

**FIGURE 1:**
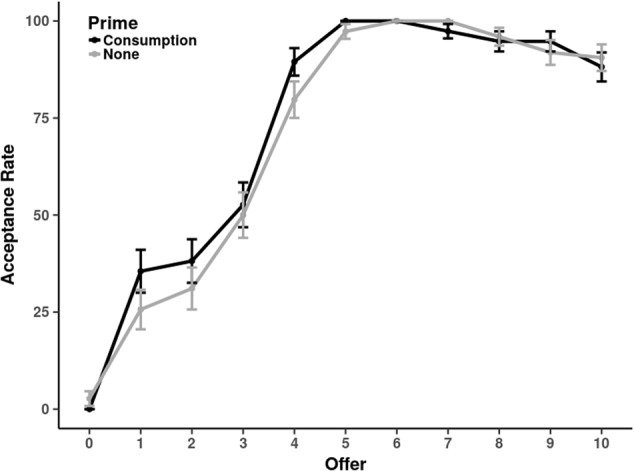
Acceptance rates (in %) for different offers (0 – 10 tokens) in Experiment 1. The black line shows the consumption-prime condition. The gray line shows the no-priming-condition.

In a more sophisticated analysis, we fitted a multi-level model using the lme4 package for R (Bates et al., 2015). Specifically, we conducted a binomial regression analysis (logit) with the decision (*reject* = 0; *accept* = 1) as dependent variable. The offer variable was treated as random effect whereas role order and priming were treated as fixed effects (dummy coding: *proposer first* = 1; *consumption prime* = 1). Most importantly, this analysis yielded significantly positive main effect of consumption priming, *β* = 2.73, *SE* = 0.62; *p* < 0.001. These results are illustrated **Figure 1**.

Moreover, the analysis revealed a main effect of role order (*β* = 1.63, *SE* = 0.63; *p* = 0.010) as well as an interaction between role order and priming (*β* = -3.00, *SE* = 0.95; *p* = 0.002). That is, responders generally accepted more offers if they played as proposers before or if they were primed with consumption. However, the significant interaction suggests that priming only has an effect if participants play as responders first, i.e., directly after the priming.

### Discussion

The proposer data provide strong evidence for the typical Ultimatum Game anomaly, which may be based on the proposers’ anticipation of the responders’ anomalous choices (Forsythe et al., 1994; Weg and Zwick, 1994). Specifically, instead of offering the smallest non-zero amount, proposers on average offered almost half of the available resources to the responders (for similar results see Güth and Kocher, 2014). As predicted, priming consumption did not have an effect on proposer decisions. In contrast, the responder data not only provide strong evidence for the typical rejections of non-zero offers (one-token offers were rejected by roughly 75% of the participants) but also support our theorizing. That is, if consumption opportunities were cognitively accessible, acceptance rates of disadvantageously unequal payoffs were increased. In fact, if participants made a decision as proposers between the priming and their responder decisions (thereby probably reducing the accessibility of consumptive opportunities) priming had no effect anymore. In sum, this evidence provides a first support for the idea that the preference for higher payoffs hinges upon the cognitive accessibility of consumption opportunities that can be realized with these payoffs.

## Experiment 2: Preference Components in Conflict

In the Ultimatum Game, the preference for higher payoffs and the preference for equal payoffs can be either aligned or in conflict. In the second experiment, we will explore this conflict in more detail. Therefore, Experiment 2 was similar to the previous one but we additionally measured reaction times as a behavioral measure of conflict. While Rubinstein (2007) suggested that in economic games, response times (RT) allow a differentiation between “cognitive” and “instinctive” decision processes (see also Rand et al., 2012), more recent research (Evans et al., 2015) interprets RTs as an indicator of conflict (see also Krajbich et al., 2014, 2015). As a result, if responders are offered positive but unequal payoffs the preference for higher payoffs is in conflict with the preference for equal payoffs which should manifest in slower decisions (Achtziger and Alós-Ferrer, 2014). Even further, if the preference for higher payoffs is strengthened by priming consumption, the conflict should be amplified and result in even slower decisions.

### Sample and Design

Two hundred and forty participants (*age: M* = 22, *SD* = 4; 79% female) were recruited on campus for a lab experiment. Because one goal of this experiment was the replication of the results from Experiment 1, we decided to increase the sample size in order to achieve sufficient power. Groups of maximally four participants completed a series of unrelated experiments (the Ultimatum Game always was the first task) on computers placed in cubicles. Participants received a show-up fee of 4€ for the entire experimental session lasting about 30 min in total. As an additional incentive for the Ultimatum Game, participants took part in a lottery in which they could win a 50€ Amazon gift voucher. Similar to the previous experiment, the amount of tokens earned in the Ultimatum Game determined the probability of winning the gift voucher, i.e., its expected value.

Participants were tested in a 2 (role: proposer vs. responder, w/i) × 2 (prime: none vs. consumption, b/w) mixed design. Each participant first played an Ultimatum Game in the role of responder and then played a second, independent Ultimatum Game as proposer.^2^ Payoffs from both games were again added up. In addition to proposer offers and responder decisions, reaction times served as dependent variables.

### Materials and Procedure

The general procedure was very similar to the previous experiment. However, the experimental manipulation differed from the previous experiment such that in addition to selecting a desired product category in the consumption prime condition, participants also specified the good they intended to buy with the gift voucher. Most importantly, however, we implemented a sequential version of the strategy method (Selten, 1967; Fischbacher et al., 2001) in order to measure reaction times for all responder decisions separately. In contrast to the previous experiment, we played the Ultimatum Game with 6-token-pies to avoid too long offer sequences. Therefore, responders were presented with a sequence of 7 offers (0–6 tokens) and made their decisions while we recorded reaction times for each offer. Offers were either presented in a descending or ascending order. After playing the Ultimatum Game as responder and proposer, participants again answered the same questions as in Experiment 1. The lottery procedure was identical to the previous experiment.

### Results

As expected, participants in the consumption-prime condition reported thinking significantly more about possible purchases (*M* = 2.05, *SE* = 0.10) than did participants not primed with consumption (*M* = 1.26, *SE* = 0.07), *t*(238) = 6.603, *p* < 0.001, *d* = 0.85.

#### Proposers

On average, proposers offered 3.22 tokens (*SE* = 0.08) which was significantly more than the smallest non-zero offer, *t*(237) = 28.632, *p* < 0.001.^3^ Moreover, proposer offers were not affected by priming consumption, *t*(236) = 0.589, *p* = 0.557, *d* = 0.076.

Reaction times in the consumption-prime condition (*M* = 10.822 s, *SE* = 0.798 s) also did not differ significantly from the control condition (*M* = 11.313 s, *SE* = 1.408 s), *t*(236) = 0.306, *p* = 0.760, *d* = 0.040.

#### Responders

**FIGURE 2:**
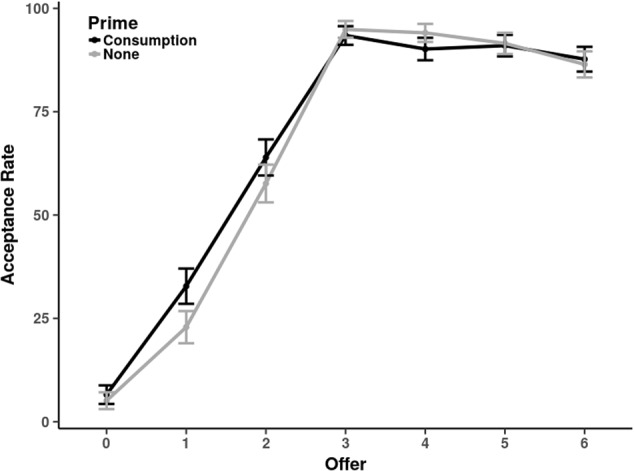
Acceptance rates (in %) for different offers (0 – 6 tokens) in Experiment 2. The black line shows the consumption-prime condition. The gray line shows the no-priming-condition.

Similar to the first experiment, we first calculated the average acceptance rates of disadvantageously unequal offers (1 and 2 token offers). A one-tailed *t*-test shows a marginally significant effect such that responders in the consumption prime condition (*M* = 0.48; *SE* = 0.04) accepted more disadvantageously unequal offers than responders in the control condition (*M* = 0.40; *SE* = 0.04), *t*(238) = 1.549, *p* = 0.06, *d* = 0.20. Moreover, we conducted the same multilevel modeling analysis as in Experiment 1. This analysis again yielded a significantly positive effect for the consumption prime (*β* = 0.48, *SE* = 0.24; *p* = 0.041). That is, responders in our second experiment also seemed to accept more offers if consumption was primed. These results are illustrated **Figure 2**.

To analyze the reaction times, we fitted another regression model with the lme4 package for R. In detail, we regressed RTs (in ms) on centered offers (squared and linear) and a dummy variable for the consumption prime.^4^ We calculated both, the random and the fixed effects for both offer variables. The consumption prime was treated as a fixed effect (dummy coding: *consumption prime* = 1). The interaction terms between both offer variables and the dummy were included as well. Degrees of freedom for the model’s parameters were approximated with the Satterthwaite procedure implemented in the lmerTest package for R (Kuznetsova et al., 2015), which also calculates *p*-values on the basis of this approximation. The statistics for the fixed effects are shown in **Table 1**.

**Table 1:** Fixed effects of responder RTs in Experiment 2.

Variable	Estimate	*SE*	*df*	*t*	*p*
Intercept	2853.92	184.69	236.69	15.453	<0.001
Offer	–48.62	106.10	232.23	–0.458	0.647
Offer squared	219.23	25.89	214.62	8.469	<0.001
Consumption Prime	492.41	258.98	236.57	1.901	0.059
Offer × Prime	25.08	149.11	233.99	0.168	0.867
Offer squared × Prime	163.08	36.76	221.3	4.437	<0.001

*All offer levels were included in this analysis.*

**FIGURE 3:**
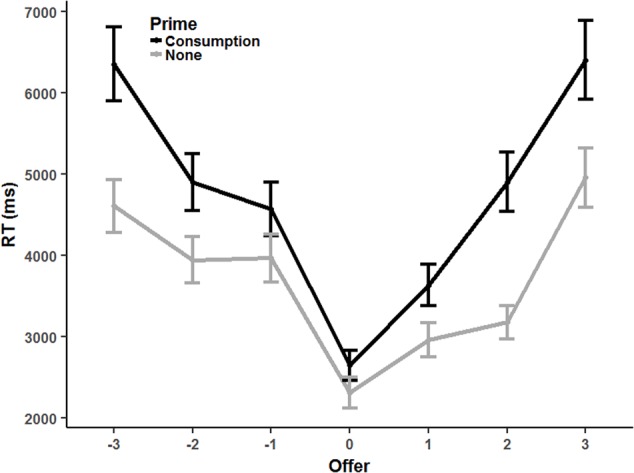
Average reaction times for different offers in Experiment 2. Offers were centered around the equal split offer (3 tokens). The black line shows the consumption-prime condition. The gray line shows the no-priming-condition.

The estimate for the offer-squared variable significantly positive which suggests a u-shaped pattern for the RTs. Furthermore, decisions are fastest for 3 token offers which can also be seen in the insignificant estimate for the offer variable. In sum, reactions are fast for equal offers but increase for both, advantageously and disadvantageously unequal offers. The results are also shown in **Figure 3**.

Moreover, the lack of either a significant main effect of consumption priming or an interaction with linear offers suggests that the apex of the parable did not change if consumption opportunities were highlighted. That is, decisions were still fastest for 3 token offers. In contrast, the interaction between offers squared and consumption-prime is significantly positive. Therefore, the u-shape is more pronounced if consumption was primed. That is, the RT difference between equal split offers and unequal split offers was larger under these circumstances.

### Discussion

The data suggest that in the second experiment, decisions of both, proposers and responders were very similar to the first experiment. That is, proposers offered more than would be traditionally considered rational but their offers were not affected by our experimental manipulation. Responders rejected unequal non-zero offers which marks the usual Ultimatum Game anomaly. But more importantly, we replicated the effect from the first experiment that more unequal offers were accepted if consumption was primed.

While the decision data further support our previous conclusions, the reaction times yield new evidence for a conflict between different preference components.^5^ As expected, when the preference for higher payoffs was aligned with the preference for equal payoffs, responders reacted faster than when both preference components were in conflict. Interestingly, even though the decisions suggest that when facing advantageous inequality, the conflict is more often resolved in favor of higher payoffs, but the increased reaction times in situations of advantageous and disadvantageous inequality suggests that the psychological processes underlying inequity aversion operate for both types of inequity. Most important, however, increasing the salience of consumption opportunities amplified the conflict. Importantly, the conflict seems to be amplified for situations exhibiting either advantageous or disadvantageous inequity. Correspondingly, the increased reaction time differences between offers leading to equal and unequal payoffs suggests that the preference for higher payoffs is at odds with the preference for equal payoffs no matter who is favored by the inequity. In sum, the effects of consumption priming on decisions and reaction times supports our hypothesis that the preference for higher payoffs relies on the payoffs’ consumptive potential.

## General Discussion

The current results support the idea that economic decision making is guided by two different preference components, the preference for higher payoffs and the preference for equal payoffs. Moreover, our novel finding that consumption priming seems to reduce responders’ rejections of disadvantageously unequal payoffs suggests that the preference for higher payoffs is rooted in the desire for consumption. In addition, our reaction time data points to an increased conflict between both preference components if consumptive consequences are salient. That is, while the preference for equal payoffs remains unaffected, the preference for higher payoffs is strengthened if the decision context includes consumptive opportunities. While “social preference” models recognize that there is considerable heterogeneity between decision makers to which degree each component contributes to the final choice (e.g., Fehr and Schmidt, 1999), our results furthermore indicate that there also is considerable heterogeneity between decision contexts. Specifically, if consumption is salient in a given context, the preference for higher payoffs should also receive higher weights in the utility functions.

Importantly, we do not consider consumption priming a feasible intervention in an applied context. Based on our results, it seems that its effect is too subtle to affect behavior outside of controlled laboratory settings. Instead, however, our results may shed light on the cognitive processes underlying the formation of preferences. Specifically, we propose to focus on the evaluative judgments performed by the decision makers. Even though this approach had already been instigated by the 19th century Austrian economist Menger (1871), judgmental processes have not received sufficient attention in the economic literature. Based on the idea that “social preferences” incorporate two distinct components, we suggest that each component is based on a specific type of evaluative judgment. That is, we propose a distinction between deductive and comparative evaluations which underlie the preference for higher payoffs and the preference for equal payoffs, respectively. In general, comparisons of an outcome with a standard were found to be ubiquitous (e.g., Mussweiler and Epstude, 2009). This also applies to the Ultimatum Game. From this perspective, the rejection of unequal offers suggests that responders may have compared the offered payoff with some reference point which in the context of this game seems to be an equal or “fair” split of the cake (Kahneman et al., 1986; Fehr and Schmidt, 1999). On the other hand, responders may also engage in an alternative evaluative judgment which is more deductive in nature. Specifically, they may evaluate their monetary payoff in terms of the goals or consumption opportunities it represents. In fact, such an evaluative strategy seems to be implied if money is equated with utility in traditional economic theorizing (Friedman and Savage, 1948). Interpreting this assumption in terms of psychological processes suggests that the value of money (or a monetary payoff) is derived from the value of its selected use.

The results from the present experiments support this theorizing regarding an interplay between different evaluative strategies. Specifically, we presented evidence that introducing information relevant for deductive evaluations promotes their corresponding behavioral implications (i.e., more offers accepted). Analyzing the decisions from both experiments,^6^ acceptance rates for disadvantageously unequal offers in the consumption-prime condition (*M* = 0.51, *SE* = 0.03) were on average 8% higher than in the control condition (*M* = 0.43, *SE* = 0.03), *t*(388) = 1.958, *p* = 0.051, *d* = 0.20. To be sure, the effect of consumption priming on responder decisions seems to be small at best and even remains slightly above the 5% threshold in the combined analysis. Nonetheless, even small effects allow conclusions about the underlying psychological processes.

In addition, previous research also supports our theorizing. Regarding the role of deductive evaluations, the results from Harlé and Sanfey (2007) could be interpreted such that the experimentally induced positive mood of the responders was used to deduce the value of the payoffs (see Schwarz, 2011) which reduced rejections of unequal offers. Furthermore, there should be fewer rejections of unequal offers, if the necessary information for the comparative judgment is not available or if the comparison itself is difficult. Indeed, some existing findings support this theorizing. Croson (1996) varied participants’ knowledge about the cake size as well as the format of the offers being either in absolute or relative terms and found that withholding the necessary information for a comparative evaluation (i.e., a standard of comparison) indeed led to lower rejection rates. In a related vein, Handgraaf et al. (2004) have shown that the acceptance rate of unequal offers increased if the payoffs of proposers and responders were different types of lottery tickets similar to the p-bets and $-bets in Lichtenstein and Slovic (1971). That is, the incommensurability of the payoffs seems to have impaired the comparative strategy.

From our perspective, the priming manipulation has caused respondents to transform the payoff’s numerically defined value into a categorically based evaluation, that is, an evaluation derived from the superordinate category an outcome is assigned to. To be sure, this transformation may be linked to a more concrete representation of the outcome. While the abstractness versus concreteness of mental representations have their own impact on human judgments (Borgida and Nisbett, 1977; Trope and Liberman, 2010), the current effect does not necessarily depend on the vividness of the outcome. Even though a vivid imagination of a consumption opportunity (“a red jacket”) may elicit positive affect which may by itself serve as information in deductive evaluations (see, Strack et al., 1985), a more abstract representation of the consumption (“something nice to wear”) may also be the basis of deductive evaluations. Of course, this is an avenue for further research.

Moreover, the present results can be interpreted such that consumption priming altered the very nature of the comparison. That is, instead of comparing payoffs numerically to reach a decision, responders might have only considered the opportunity costs of rejecting inequity if they were represented as forgone consumption.^7^ In fact, previous research indicates that opportunity costs are often neglected if they are not mentioned explicitly (Frederick et al., 2009; Spiller, 2011). However, so far it is unclear whether opportunity costs were perceived to be higher if consumption was salient or if the priming made it more likely that they were considered at all. Future research is necessary to address this question.

More generally, we deem it important to distinguish between *process* models and *as-if* models^8^ of economic behavior (see Friedman, 1953). During the past years, the predictive power of as-if models has been greatly increased by introducing extended utility functions (as in “social preference” models) but this approach deliberately abstracts from the actual psychological processes underlying decision making (see Bruni and Sugden, 2007). Thus, our exploration of the cognitive operations underlying economic decision making might raise the question why we should at all pay attention to psychological processes in economic theorizing? Our answer is twofold. First, process models are able to integrate a wide range of seemingly unrelated, anomalous findings whereas each anomaly entailed its own as-if model featuring utility functions tailored to its specific context. In contrast, Simonson’s (2008) extensive literature review of findings on unstable and inconsistent preferences concluded that people often gravitate toward comparative evaluations if the context provides the relevant information but may also evaluate goods on the basis of “non-quantitative aspects (e.g., the taste of beef jerky, a motion sensitive videogame remote)” (Simonson, 2008, p. 20). Thus far, however, both types of judgments have not been treated as distinct psychological processes that may interact in economic decisions.

The second advantage of process models over as-if models is their ability to generate policy advice that goes beyond interfering in the price system (e.g., via taxes). Because as-if models give no insight into the actual decision processes but only describe how price changes translate into to changes of behavior, any advice derived from them must necessarily involve changes in prices. While such traditional interventions still form the core of economic policy, new methods of directing economic interactions into socially more desirable paths become increasingly important. Most prominently, Thaler and Sunstein (2008) argued that “nudging” people is not only often more effective than traditional interventions but also far less costly. As a consequence, even the World Bank advocated using “nudges” to solve society’s most pressing economic problems (World Bank, 2014). However, to develop new and better “nudges” it is paramount to analyze and better understand the psychological processes that govern our economic behavior.

In sum, the proposed duality of evaluative strategies may be a starting point for a more general conceptual framework that integrates both the “rational” model and the implications of its anomalies. As a consequence, anomalous deviations may become less important as mere illustrations for the shortcomings of homo economics (Levine, 2012). Instead, they may become testing grounds for any theorizing that takes a closer look at the psychological processes underlying economic behavior (see also Crusius et al., 2012).

## Ethics statement

No ethics approval was required for our experiments. We only used standard paradigms which do not entail any risks for the human subjects. Subjects were invited to participate and received a short description of the experiment. All information provided to the subjects was true. We did not obtain informed consent in written from but participants indicated their informed consent performatively by starting the experiment. Also, subjects could abort the experiment at any time.

## Author Contributions

Both authors have contributed equally to the research presented in this paper and both approved it for publication.

## Conflict of Interest Statement

The authors declare that the research was conducted in the absence of any commercial or financial relationships that could be construed as a potential conflict of interest.

## Footnotes

1 For two reasons, decisions in both roles were analyzed separately. First, based on our theorizing, we hypothesized that consumption priming only affects responders’ decisions. Second, responses in both roles are qualitatively different. That is, proposers choose between eleven options (offer 0 – 10 tokens) while responders make eleven binary choices. Hence, we did not test the interaction effects of consumption priming and role.

2 Due to our main interest in responder decisions and the results from Experiment 1, we decided to always let participants play as responders directly after the priming.

3 Two participants had to be excluded from this analysis due to offering more than the total endowment.

4 RTs faster than 150 ms and slower than 3 SDs above the mean were excluded from this analysis. However, including these outliers does not qualitatively change the results.

5 To some degree it, is surprising that responders show the highest reaction times for the most extreme offers. After all, there should be no conflict between both preference components for zero offers. However, such an extremely unequal offer might be very unexpected and thereby elicit surprise. Being offered the entire amount might then be similarly surprising. Nonetheless, we argue that the surprise is based on the very preference components we discuss in this paper. Specifically, the surprise reaction is driven by receiving an unexpectedly unequal offer which is reflected in slower reactions. In addition, if consumptive opportunities are made salient, more information has to be integrated with the surprising outcome which leads to even slower reactions. To be sure, these interpretations are rather speculative but because our results remain very much the same, if we exclude the most extreme offers, we deem our interpretation of the reaction time data in terms of conflict between two distinct preference components the most parsimonious one.

6 Even though standardized mean differences across all offers suggest that the effect of consumption priming on acceptance rates are of similar size in both experiments, the more sophisticated regression analyses indicate that the priming effect was smaller in Experiment 2. This either points to the inherent statistical variations of observed effect sizes in priming research or it may reflect the changes we made to the experimental paradigm for Experiment 2. In any case, the exact effect size of our priming manipulation requires future research.

7 To a certain degree, responders who had been primed with consumption may also have experienced the rejection of positive offers as a loss. This idea, which has been formulated by one of our reviewers, seems to be an interesting avenue for future research.

8 “Consider the density of leaves around a tree. I suggest the hypothesis that the leaves are positioned as if each leaf deliberately sought to maximize the amount of sunlight it receives […] the hypothesis does not assert that leaves do these things but only that their density is the same *as if* they did. [...] It is only a short step from these examples to the economic hypothesis that under a wide range of circumstances individual firms behave *as if* they were seeking rationally to maximize their expected returns (generally if misleadingly called “profits”) and had full knowledge of the data needed to succeed in this attempt; *as if*, that is, they knew the relevant cost and demand functions, calculated marginal cost and marginal revenue from all actions open to them, and pushed each line of action to the point at which the relevant marginal cost and marginal revenue were equal” (Friedman, 1953, p. 12–13).
